# Supplementary material for: Are carbon emissions trading and green financial instruments synergistic? -Comprehensive quantitative research based on content analysis
Source: PLoS One. 2024 Mar 7;19(3):e0298601. doi: 10.1371/journal.pone.0298601 (PMC10919601; doi:10.1371/journal.pone.0298601)
Supplement: S1 Data — (ZIP) [file pone.0298601.s001.zip › Data/地级市Z_F工作报告.zip!/╣π╢1⁄2/╓Θ║ú╩╨/└┤╫╘╣1⁄2╓┌║┼í╛┬φ┐╦╩2╛▌═°í┐_2008.docx]

各位代表：

现在，我代表市人民政府向大会作政府工作报告，请予审议，并请各位政协委员和其他列席人员提出意见。

一、２００７年工作回顾

２００７年是我市经济社会全面快速发展的一年，也是珠海人民得到更多实惠的一年。市政府在省委、省政府和市委的正确领导下，在市人大、市政协的监督支持下，坚持以邓小平理论和“三个代表”重要思想为指导，认真贯彻落实科学发展观，不断完善发展思路，大力优化发展环境，着力推进经济发展，致力保障和改善民生，积极推进和谐社会建设，有力地推动了我市经济社会全面进步。

（一）经济保持快速发展，综合经济实力进一步增强。

经济增长速度加快。全市实现本地生产总值８８６．８亿元，比上年增长１６．５％，为２００４年以来的最快发展时期。人均生产总值６．１万元，增长１５％。城镇居民人均可支配收入１９２９０元，增长９．２％。工业经济加快发展，工业总产值突破２５００亿元，增长１８．７％；工业增加值４７１．９亿元，增长２０．１％。第三产业贡献率同比提高２．７个百分点。编制了旅游发展总体规划，加快推进旅游业发展，海泉湾成为全国首个“国家旅游休闲度假示范区”。投资、出口、消费对经济增长的拉动作用明显，完成固定资产投资３４３．２亿元，增长３３．４％；社会消费品零售总额３０１．５亿元，增长１８％；外贸出口总额１８４．７亿美元，增长２４．５％。金融机构各项存款余额１３８８亿元，比年初增长２０．８％；各项贷款余额６８３．２亿元，比年初增长２９．５％。西部地区工业发展迅速，经济增速高于全市２．１个百分点。

经济质量和效益明显提升。工业适度重型化趋势明显，优势产业加快发展。电子信息、家电电气、电力能源、生物医药行业产值增幅均超过２０％，轻重工业产值比例由上年的４１．２∶５８．８调整为３９．５∶６０．５。打印耗材产业集聚效应明显，产量约占全国八成，全国唯一的国家打印耗材质检中心落户珠海。财政收入和企业效益快速增长。来源于珠海的财税收入总量由上年的１９５．２亿元增加到２４８．９亿元，其中，地方财政一般预算收入７５．８亿元，净增１５．５亿元，增长２５．８％。规模以上工业企业利润总额增长７０．８％，同比提高４６．３个百分点。

（二）基础设施建设实现新突破，城市环境呈现新面貌。

基础设施建设取得重大进展。建立了市级领导分工协调重大项目工作机制。以“一桥双港三铁四高速”和“六横八纵”为骨架的立体交通体系规划建设成效显著，港珠澳大桥项目融资方案基本确定，广珠铁路前期工作顺利推进，广珠城际轨道交通建设全线推进，江珠高速公路建成通车，新建的南琴路和改造的１０５国道上冲段、人民西路、情侣路等道路竣工通车。洪湾港一期工程竣工，前山港搬迁完毕，高栏８万吨级石化码头试产营运，两个５万吨级集装箱码头、液化天然气项目一期工程等重大项目加紧建设。珠海电厂３、４号机组投产，５００千伏珠香线建成通电。咸期应急供水工程顺利推进，第三条供澳门原水管动工建设。

市容市貌明显改善。实施“１３５”行动计划成效明显。改造和美化了主城区４７条人行道、２２条横街小巷，修通了９条、亮化了４９条城市道路，建设和改造了２５个街心公园，建成前山河东岸景观带、野狸岛公园。加大整治违法建设力度，拆除违法建筑面积４３万平方米。集中整治农贸市场环境和“乱摆卖”行为初见成效。完成前山河综合整治首期工程和白沙河综合整治工程，建成南区、井岸污水厂和三灶污水处理设施，实施香洲截污分流改造工程，启动前山、拱北片区和新青等污水处理设施建设，西坑尾垃圾填埋场投入使用。在经济发展的同时，生态环境保持良好，荣获省环保责任考核“六连冠”和珠江综合整治考核“三连冠”。

（三）改革开放深入推进，开放型经济快速发展。

体制改革不断深化。国企产权多元化改革和引入战略投资者取得新成效。格力集团、珠光集团和市商业银行重组工作顺利推进。引进中石油集团投资城市管道燃气项目，推动城市管道燃气特许经营改革。平沙、红旗和白藤农场改革加紧推进，有效化解了农场金融债务。

扩大开放取得新成绩。对外贸易规模扩大，进出口总额３９８．７亿美元，增长２１．５％。积极开拓对外交往新渠道，成功举办珠澳投资环境推介暨经贸交流会等活动，招商引资成效明显，项目质量有所提高。实际利用外商直接投资１０．３亿美元，增长２４．８％。新批外商直接投资项目７６１个，其中，世界５００强新设和增资项目３０个，同比增加１４个。外经贸工作成绩突出，获得国家和省政府授予１０个奖项。内源型经济发展加快，引进内资注册资本金总额２１．９亿元，增长５８％，其中１０００万元以上的项目７４个，同比增加４２个。

口岸和港澳工作得到加强。强化口岸和涉港澳事务管理，促进了珠港澳交流与合作。加快口岸规划建设，创新查验模式，优化通关环境，建成和启用横琴口岸新旅检大楼，开通九洲港至香港国际机场海上新航线，口岸通关效率提高，获省政府授予口岸大通关建设一等奖，口岸联检单位获得国家和省授予的６个奖项。全年珠海各口岸出入境人数达到８４３６．５万人次，增长５．７％，出入境交通工具２８８万辆（艘）次，增长１０．８％。珠港机场合作成效明显，珠海机场年客运量首次突破１００万人次，增长３０．３％。港口集装箱吞吐量创近年来新高，增长１９．５％。珠澳跨境工业区建设稳步推进，配套基础设施建设与项目招商进展顺利。

（四）自主创新取得新成效，科技事业持续发展。

企业自主创新能力提高。创新型企业不断发展壮大，经省认定的高新技术企业和软件企业分别增加到２２５家和１９４家，蓉胜超微等４家高新技术企业成功上市。科技创新取得丰硕成果，全市专利申请量２２１７件，增加１８７件，１０项专利技术被纳入广东省专利技术实施计划，９个项目获国家技术创新基金支持，金山软件公司“文字操作系统办公软件２００５”项目荣获国家科技进步二等奖。新增广东省“名牌名标”１４个，国家和省级“名牌名标”达到７７个。三灶镇、井岸镇成为广东省技术创新专业镇。全市高新技术产品产值９５０亿元，占工业总产值的３８％，高新技术产品出口占全市出口总额的４３．９％；软件产品产值１２０亿元，增长３３％。我市连续第四次被评为“全国科技进步先进市”。

科技创新服务体系逐步完善。落实激励自主创新的配套政策，依托大学园区和国家高新区，积极推进产学研结合，珠海被列为“广东省教育部产学研结合基地”示范市，高新区被列为国务院侨办“引智引资重点联系单位”。目前，全市有３家国家重点实验室分支机构、３家国家级工程技术研发中心、１个国家“８６３计划”成果转化基地以及７９家省级和市级企业工程、技术中心，建设集成电路设计等９个科技创新公共服务平台，自主创新成为我市转变经济发展方式的重要动力。

（五）支农惠农力度加大，新农村建设稳步推进。

农村“三化”进程加快。全市财政安排“三农”投入１４．７亿元，实现农业总产值３８．２亿元。农业普查和２０个新农村建设试点村规划基本完成。农村工业化进程明显加快，５个镇工业产值超百亿元。工业园区建设带动了农村城镇化发展，改造了一批农村公路、危桥、乡镇渡口，珠峰大道改造竣工通车。城乡水利防灾减灾工程加紧推进，加固海堤５３公里，新建水闸３８座。农业产业化步伐加快，实施农业园区带动战略，规划建设４个农业园区。新组建１９家农民专业合作社，新评定８家市级农业龙头企业，３家企业被认定为省重点农业龙头企业。加快“菜篮子”工程建设，建成市农副产品批发中心。加强农产品源头监管，农产品质量安全监测合格率居全省前列。农村民主法治水平得到提高，斗门区南潮村成为“全国民主法治示范村”。

农民生活水平不断提高。农渔民人均纯收入７６２２元，增长８．８％。三灶鱼月村、鱼林村集体年收入超过一千万元。１１３个农村卫生服务中心立项建设，其中２５个试点单位已投入使用。农民养老保险参保人数达到９．４万人。大力改善农村人居环境，市投入６２０５万元改造华侨农场危房，新开工建设１８０８户；完善农村地区供水管网；增加农村地区公共汽车班次和线路，新建一批候车亭，方便农村群众出行；１５条生态文明示范村通过考核验收。

（六）社会事业加快发展，人民得到更多实惠。

改善民生取得突出成效。公共财政对民生投入明显加大，全市财政安排教育、医疗卫生、社会保障和就业、文化体育、环境保护、农林水事务等六项支出达３４亿元，占财政一般预算支出的４１％。１４．６万名珠海户籍的中小学生享受十二年免费教育，全年免交学杂费和书本费７７１５．７万元。新建、扩建１０所中小学校，公办中小学校新增学位４２１７个，北师大（珠海）附中等学校竣工开学。社会保险参保人数超过２８０万人次，未成年人医疗保险突破１６万人。市计生服务中心和市妇幼保健院改扩建工程竣工启用。提供免费婚前和孕期医学保健服务，并将外来女工纳入免费孕检范围。试行“大病统筹救助、中病进入保险、小病治疗免费”的全民医疗保障制度，并将外来务工人员纳入医疗救助范围，其中“小病治疗免费”在农村整体推进。调整医疗服务价格，医药费用下降３．２％。我市被列入国家统筹城乡就业试点城市，新增就业岗位约３万个，４４２８名农村青年参加职业技能培训，一万多名农村富余劳动力转移就业。再就业率达到６６．９％，完成省下达任务的１１５％。城镇登记失业率为２．７６％，完成省下达的控制在３％以下的目标。超过１６万人次得到低保资金救济。领取老年津贴人数近３万人。残疾人社会保障、康复和就业服务等工作得到加强，一万多名残疾人受惠。开展红十字会博爱专项医疗救助活动，对先天性心脏病儿童、白内障老人和孤儿实施全面救治与救助。完善政府廉租房制度，统筹一批公产房作为廉租房，为７６３户住房困难低保户发放租赁住房补贴，多渠道解决了主城区一批低保户的住房问题。６０岁以上长者乘坐公共汽车享受免费或优惠政策。城乡居民享有的公共服务明显增多，更多地让广大市民分享到改革发展的成果，荣获“中国十大最具幸福感城市”称号。

“平安珠海”建设成效显著。维稳工作成绩突出，为改革开放和经济建设营造了良好的环境。平安和谐社区创建工作深入开展，公安“三基”工程建设扎实推进，治安秩序良好，刑事发案连续五年下降，其中“两抢”案件同比下降３０．２％，我市被评为“全省社会治安综合治理优秀市”。打击走私保持高压态势。率先在全省组建专职人民调解员队伍，建立行政司法联动调处欠薪工作机制，社会矛盾排查调处能力得到加强。健全市、区应急管理网络，应急处置能力大大提高。安全生产形势保持稳定，全年事故宗数、死亡人数、受伤人数和直接经济损失同比分别下降２７．５％、８．１％、３２．８％和３７．３％，消防事故实现“零死亡”。海上搜救能力进一步提高，配备两架国内最先进的直升机，搜救成功率达９３．６％。全面推行建筑工人“平安卡”管理制度。产品质量和食品安全专项整治通过国家和省组织的检查验收。我市被国家权威机构评为“中国十大和谐名城”。

文化体育事业加快发展。创建“全国文明城市”各项工作进展顺利。加大力度建设公共文化设施，古元美术馆建成开放，市档案馆新馆即将竣工，基层文化站建设取得可喜进展。文化遗产保护力度加大，唐家湾镇被列入“中国历史文化名镇”，咸水歌等四种民俗艺术列入广东省非物质文化遗产代表作名录，苏曼殊故居等文物保护单位动工修缮。成功举办第一届民间艺术大巡游，群众性文化活动和高雅艺术演出丰富多彩。文化产业加快发展，南方文化产业园等重点文化产业项目稳步推进。协助国家文化部成功承办“第三届尼泊尔中国节”。群众性体育活动蓬勃发展，市体校一期工程基本竣工，成功举办Ａ１世界杯汽车大奖赛等系列赛事，珠海籍运动员在第六届全国城市运动会上取得７枚金牌的好成绩。

（七）政府自身建设不断加强，行政效能逐步提高。

依法行政得到加强。自觉接受市人大及其常委会的监督，主动接受人民政协的民主监督，认真听取民主党派、工商联、无党派人士、人民团体意见，通过多种形式征求专家学者和人民群众的建议，接受新闻媒体和社会各界的监督。提请市人大常委会审议地方性法规草案７件，已颁布３件；提请审议专项工作报告５项，及时反馈研究处理情况。制定政府规章３件。高度重视市人大代表、政协委员的意见和建议，办理代表建议１９１件、政协提案３２２件。推进民主科学决策，注重改进政府议事规则和办事程序，深入推进政务公开，完善重大事项公示、听证和决策专家咨询制度，提高了工作透明度，促进政府运作的制度化、规范化、科学化。

政府建设取得新成效。建立部门行政首长问责制，推行部门责任白皮书制度，启动决策督查督办与行政效能监察联动机制。加强行政服务中心建设，创新政府公共服务平台，推广应用公用审批平台、网上审批系统、行政效能电子监察系统、电子督办网络和外经贸业务电子公章系统，有效提升行政效能，全年审批和服务事项办结率达９７％以上。深入开展治理商业贿赂专项工作，继续开展机关作风建设和民主评议政风行风活动，加强行政监察和审计监督，政府自身建设取得了新的成效。

社会各项事业有了新的发展。双拥工作得到加强，连续第六次获得全国“双拥模范城”荣誉称号。人口计生工作成绩突出，以优良成绩完成了省下达的任务。中央和省驻珠各单位在我市经济社会发展中充分发挥作用，新闻出版、民族宗教、外事侨务、对台、海防、人防、信访、档案、气象、仲裁、地方志、港澳流动渔民等工作得到加强，老龄、福利和慈善事业健康发展。

在保持经济社会快速发展的同时，想方设法解决一批在发展中遗留的问题。妥善化解城中旧村改造、企业债务等矛盾，加强政府债务管理，全年偿还政府工程款及历史债务１２．２亿元，按合同偿还银行贷款本息６．４亿元，有力地促进了社会稳定和谐。

各位代表，回顾２００７年的工作，我们深感成绩来之不易！在此，我代表市人民政府，向奋斗在各条战线的全市广大干部群众，向各位人大代表、政协委员、各民主党派、工商联和社会各界人士，向中央和省驻珠单位、驻珠军警部队官兵，向为珠海发展作出贡献的老领导、老同志，向所有关心和支持珠海发展的港澳同胞、台湾同胞、海外侨胞和国际友人，表示崇高的敬意和衷心的感谢！

在看到我市经济社会快速发展的同时，也要清醒地认识到，我们的工作与全市人民的期待还存在不少差距，与争当实践科学发展观排头兵的要求还有较大距离。主要是：在思想观念方面，特区的改革创新锐气有所弱化，开拓意识不够强；在经济运行方面，物价结构性上涨偏快，固定资产投资结构不够合理，产业发展缺乏新的龙头带动，竞争力不强，第三产业比重不高，节能减排任务还很重；在园区发展方面，总体上仍未摆脱粗放型发展模式，园区基础设施建设仍然滞后，产业特色不明显、优势不突出，单位面积产能和效益都不高；在自主创新方面，创新能力不强，企业技术研发投入和创新型人才不足，高新技术产业发展不快、效益不高；在科学规划方面，未能发挥规划先导作用，规划滞后于经济社会发展需求，城市规划和产业发展规划亟待加快；在统筹城乡方面，区域发展不平衡，城乡差距较大，镇村经济实力比较薄弱，农民增收渠道不多，农民财产性收入和就业性收入比例不高，统筹城乡发展力度有待加大；在城市管理方面，违法建设、违法装修和影响市容市貌等现象和行为屡禁不止，尤其是城乡结合部乱搭乱建现象比较严重，影响了城市科学发展；在政府自身建设方面，管理创新有待加强，在某些领域管理制度还不够完善，社会管理和公共服务能力有待提高；在作风建设方面，一些政府部门职责重叠交叉、相互推诿扯皮、办事效率低下，一些工作人员服务意识和责任心不强、服务水平不高，有的甚至“吃拿卡要”，以权谋私，贪污腐败，在群众中造成了极坏影响，等等。对此，我们一定要始终保持清醒头脑，增强忧患意识，以对党、对国家、对人民高度负责的态度，采取有效措施，切实加以解决。

二、２００８年工作安排

今年是落实党的十七大精神的重要一年，又是我国改革开放３０周年，也是珠海经济特区成立２８周年，做好今年的工作意义十分重大。要顺应新形势，适应新要求，以世界眼光和战略思维审视未来、谋划发展，要坚持好字优先、又好又快，走珠海特色的科学发展道路。要大力弘扬敢想敢干、敢闯敢试、敢为人先的特区精神，更加注重转变发展方式、提高经济质量，更加注重推进重大交通设施建设、夯实发展基础，更加注重节约资源和节能减排、保护生态环境，更加注重推动重大项目建设、增强发展后劲，更加注重改善民生、促进社会和谐，更加注重政府自身建设、建设人民满意的服务型政府，以新一轮的思想大解放推动新一轮大发展。

今年市政府工作总的思路是：高举中国特色社会主义伟大旗帜，坚持以邓小平理论和“三个代表”重要思想为指导，全面落实科学发展观，认真贯彻落实中央、省和市委的一系列重大部署，继续解放思想，坚持改革开放，坚持科学发展，坚持以人为本，充分发挥自身比较优势，全面实施“以港立市”、“工业强市”、“科教兴市”和“三产旺市”战略，着力建设大交通、着力发展产业集群、着力拓展城市空间、着力构建和谐社会，推动经济社会跨越式发展，争当实践科学发展观排头兵。

今年我市经济社会发展的主要预期目标是：本地生产总值增长１５％，总量超过１０００亿元，人均增长１０％；单位生产总值能耗下降２．９％以上；二氧化硫排放量、化学需氧量分别控制在２．６万吨和２．５万吨以下；工业增加值增长１７％；固定资产投资增长１９％；社会消费品零售总额增长１５％；外贸进出口增长１３％；实际吸收外商直接投资额增长１３％；引进内资注册资本金总额增长２０％；财政一般预算收入增长１６％；城镇登记失业率控制在３％以下；城镇居民人均可支配收入增长６％；农渔民人均纯收入增长７％；人口自然增长率控制在７．８‰左右。

完成今年各项任务，要着重抓好以下工作：

（一）切实转变经济发展方式，推动经济又好又快发展。

加快现代产业体系建设。加快产业结构调整升级，大力发展优势产业，壮大产业规模，逐步形成以高新技术产业为先导、先进制造业为主体、基础产业为支撑、服务业全面发展的产业格局。大力实施“工业强市”战略，坚持产业集群化发展，充分发挥龙头项目的带动作用，培育现代产业集群和特色产业，着力打造以格力为龙头的家电电气产业集群、以伟创力为龙头的电子信息产业集群、以高栏港石化基地为龙头的石化产业集群、以三灶生物医药专业镇为龙头的生物医药产业集群、以金山软件为代表的软件产业集群、以炬力为代表的集成电路设计产业集群、以天威飞马和纳思达为代表的打印耗材产业集群，提高产业集聚能力，延长产业链条，着力提高我市产业的国际竞争力。

引导区域产业合理布局。以编制实施主体功能区规划为契机，充分发挥区域比较优势，促进生产要素合理配置，形成功能优势互补、区域产业布局科学的发展新格局。香洲主城区重点发展高新技术、现代服务业等产业，金湾区重点发展港口物流、重化工业、先进制造业、旅游业、会展业等优势产业，斗门区重点发展先进制造业、现代农业和生态旅游业等产业。

提高园区发展水平。经济功能区是发展新型工业和现代服务业的主战场，要大力发挥功能带动作用，找准定位，发挥优势，建设产业园区。要完善规划，加快编制和完善产业发展规划、园区分区规划、控制性详细规划和土地利用规划。要加大投入，落实工业园区配套资金，支持园区加快基础设施建设，提升配套水平和竞争力。要强化服务，落实简政强区措施，促进政府服务向园区和项目延伸。要加强考核，落实园区经济发展考核奖励办法，提高园区发展水平，做大做强园区产业。

大力发展现代服务业。实施“三产旺市”战略，大力发展旅游会展、商贸物流、信息服务和中介服务业等。树立“大旅游”观念，积极开展“浪漫之城？幸福珠海”旅游年系列活动，提升珠海旅游品牌。加快发展温泉、海岛、游艇俱乐部等特色旅游产品，大力推进海泉湾项目建设，加快香洲游艇码头建设。要认真总结办展经验，把第七届中国（珠海）国际航空航天博览会办出水平、办出特色。要继续办好国际赛车、国际游艇展等国际赛事和展会。鼓励发展商贸物流业，进一步完善物流业发展规划，构建物流公共信息平台。要鼓励发展信息服务业，加快发展以信息技术为核心的服务外包业，积极发展软件服务、教育培训等配套服务业。采取有效措施，大力发展总部经济。稳步推进市区旧工业小区和楼宇改造升级，扩大现代服务业的发展载体。落实国家房地产调控政策措施，进一步规范房地产市场秩序，建立房地产信息系统，促进房地产业有序健康发展。

大力发展海洋经济。充分发挥海洋资源优势，大力推进海洋综合开发，重点发展中转仓储、滨海旅游、海洋渔业和海洋生物医药产业。要积极引进大型中转仓储和豪华邮轮项目，加快九洲岛、东澳岛等近海岛旅游开发。加快海岛水库、码头、垃圾和污水处理设施建设，探索海洋新能源利用，改善海岛生产生活条件。推进人工鱼礁建设，改善近海生态环境，切实保护海洋资源。

（二）以港口开发和交通建设为突破口，大力实施“以港立市”战略。

大力发展临海临空产业。海港空港是我市发展的战略性资源。要依托港口优势，大力发展重化工业、先进制造业、物流业和航空产业。要充分利用广珠铁路建设的有利时机，鼓励有实力的中外企业积极参与港口开发建设和物流发展。大力发展以外向型、大物流为特征的临港工业，支持港口企业拓展市场，积极发展保税仓储业务，构筑现代港口物流体系，加快港口经济发展。充分发挥重大项目的带动作用，重点抓好高栏港码头仓储区、石化区、精细化工区、能源钢铁和装备制造区建设，加快壳牌润滑油、茂盛海洋石油服务、中铁武桥重工和斗门佳粤船厂等项目建设。积极争取国家和省有关部门支持，加紧落实大型炼化、ＰＴＡ三期、珠海电厂５、６号机组等重大项目的报批工作。落实航空产业园发展规划，加快园区管理体制、开发模式以及配套政策的研究，高水平推进航空产业园建设。深化珠海机场与香港机场合作，加快推动机场配套设施建设，拓展机场客货运市场，充分发挥珠海机场连接港澳机场服务珠三角西岸城市作用。

加快港口基础设施建设。理顺港口管理体制，整合资源，全力推进港口基础设施建设，进一步完善港口功能，提升我市区位优势和竞争力。加紧完善高栏港区控制性详细规划，开展万山港区研究。重点推进高栏港区东大堤等工程建设，力争两个５万吨级集装箱码头建成投产，抓紧四个１０万吨级集装箱码头、１０万吨级煤码头、１５万吨级矿石码头和３０万吨级油码头等项目的前期工作，加快桂山１０万吨级油码头改造。加强港口岸线管理，合理利用岸线资源，促进港口可持续发展。

完善双港集疏运系统。积极拓宽双港服务腹地，大力完善集疏运系统。全力推动高栏港和珠海机场对外交通网络建设，打通两港与国家交通干线、珠三角城市路网和内陆腹地之间的快速交通通道，促进陆海联运、江海转运、双港联动。要全力推进广珠铁路、广珠城际轨道等重大交通项目建设，加快推进连接高栏港区的珠港大道、珠海大道和通向珠海机场的省道２７２线的改造，加快机场北路至西部沿海高速公路的高速公路规划建设，尽快终结港口无铁路、机场无高速公路的历史。抓紧完善市内“六横八纵”快速干道交通网络规划。推进金港路规划建设，打通通往西部地区第二通道。加紧规划建设太澳高速公路珠海段，全力推进金凤路、翠屏路等路段建设，加快推进井岸二桥项目。规划建设环城城市轨道，重点推进金海大桥规划建设，进一步完善双港快速路网系统。

（三）继续实施“１３５”行动计划，拓展城市发展空间。

积极拓展新城区。要按照建设“最适宜居住、最适宜创业、最富有魅力”的滨海山水城市目标要求，抓紧第五轮城市总体规划修编，同时做好市级土地利用总体规划大纲修编和镇级土地利用规划编制工作。开展金湾区城市发展总体规划等５６项规划编制工作，加快完成唐家湾新城、西湖城区空间布局规划和控制性详细规划，推进城区“北扩、西拓”，拉开城市发展框架，拓展发展空间，以科学的规划指导城建工作，建设高品位城市。重点加快唐家后环、横琴、鹤洲南垦区等土地整合开发，按市场化原则，引入有信誉、有实力的大型企业参与新城区土地一级开发，高水平推进唐家湾新城、南湾城区、西湖城区、斗门新城、温泉新城的开发建设。

提升中心城区品位。进一步完善中心城区控制性详细规划，严格依法审批建设项目，严禁建设遮山、挡海项目，保护和增强中心城区的特色和吸引力。要进一步扩大城区绿地面积，提升园林生态城市建设水平。有计划地推进主城区快速路网升级改造，落实公交优先，解决行人安全、交通拥堵和停车难等问题。全面推进前山河和主城区排洪渠综合整治，控制陆源污染物排放。稳步推进城中旧村和旧住宅小区改造，进一步改善人居环境。

提高城市管理水平。加大城市管理和城管执法力度，大力整治违法建筑和室内违法装修等行为，规范和美化户外广告。提升物业管理水平，维护业主合法权益。加大城市环境综合整治力度，重点整治乱摆卖、乱张贴和占道经营等违法行为，狠抓农贸市场环境整治，改善居民购物环境。积极探索适应现代城市发展的城管体制，加强对城管工作的组织、投入、监督和考核，全面提高城市管理和城管执法水平。

（四）促进人口资源环境相协调，提高城市可持续发展能力。

着力抓好节能减排。强化绿色ＧＤＰ理念，绝不以牺牲环境为代价发展经济，全面推进科学发展。落实节能减排约束性任务工作责任制，保障节能减排资金投入，加快建立节能减排的统计、监测和考核指标体系，确保完成节能减排目标任务。严把项目准入关，制定投资项目节能减排评估和审查规范。推行清洁生产，大力发展环保产业。推动企业加快节能降耗技术改造，继续实施“重点耗能单位节能行动”。制定建筑节能实施办法。加快实施企业脱硫工程，开展工业固体废物综合利用和再生资源循环利用，做好循环经济试点工作。加快供电基础设施建设，提高能源保障水平。

提高土地利用率和产出率。加强土地利用管理，依法利用土地。要坚决改变土地粗放使用现象，促进节约集约使用土地。严格落实土地使用权招拍挂出让制度，严格执行闲置用地处置政策，鼓励按规划充分利用地上地下空间，鼓励企业提高工业用地容积率，提高土地利用率和产出率。加强基本农田保护，加强土地执法监督，严查土地违法违规行为，促进土地的可持续利用。

提高城乡供水安全保障能力。要加强水资源保护流域合作、合理利用水资源，加快中珠联围等城乡水利防灾减灾工程和河道畅通工程建设。建设竹银水库系统工程、南区水厂和黄杨泵站及其配套工程，切实提高我市城乡和对澳门供水安全保障能力。

加强生态环境建设。大力开展绿色创建活动，使我市山更绿、水更清、天更蓝。全力推进生态城市建设，积极迎接国家环保模范城复查。严厉查处乱挖山取土行为，要按照“谁主管谁复绿、谁破坏谁复绿”的要求，对已破坏的山体尽快完成绿化美化工作。规划建设湿地生态公园。大力推进江河水整治和治污保洁工程，加紧实施生活垃圾、医疗垃圾、污水污泥无害化处理系列工程，加快西部地区环保基础设施建设，启动万人以上镇污水厂和市环境污染监测系统工程建设。完善应急预案，提高危险品污染事故应急处置能力。

加强人口与计划生育工作。加强计生队伍建设，完善流动人口计划生育管理服务体系，稳定低生育水平，将人口出生率控制在１０．９‰以下。完善户籍管理制度，严格控制人口，优化人口结构，提高人口素质，促进经济与人口资源环境协调发展。

（五）坚持改革开放，不断增强特区发展动力。

全面推进新一轮改革开放。要认真总结改革开放３０年的实践经验，继续解放思想，坚持改革开放，大力弘扬特区精神，大力营造鼓励创新、支持改革的良好氛围，凡是不符合科学发展的体制、机制、管理方式都要改革调整，破除加快发展的各种约束和障碍，继续发挥改革“试验田”和对外开放“窗口”作用，增创发展新优势。

推进重点领域的改革。要不断深化改革，大力推进财税、金融、投资体制和国企等领域改革。贯彻执行新企业所得税法，强化综合治税，加强非税收入管理。扩大公共财政覆盖面，重点加大对西部地区的财政转移支付力度。深化预算制度改革，完善部门统一预算和国库集中支付系统，强化重点支出项目绩效评价。加强财政支出管理，推行公务卡结算改革，推进“金财工程”建设。优化金融生态环境，鼓励金融产品创新，提高金融业竞争能力和服务水平。积极支持市商业银行重组。深化农信社改革，发挥其在新农村建设中的信贷主渠道作用。鼓励企业利用资本市场发展壮大。修订政府投资项目管理条例，规范投资管理，提高政府投资效益。坚持国有企业在投资领域有进有退，努力走出一条在进中寻求发展、增强实力，在退中引入投资、壮大产业的发展路子。深化国企产权制度改革，引入战略投资者，完善企业法人治理结构。加快公汽、供排水等公益性和垄断性企业特许经营制度改革。充分发挥公共资源交易平台在资源市场化配置中的作用，发展生产要素市场，规范行业协会和市场中介组织行为。推进保税区功能整合和政策叠加试点。进一步完善“区镇合一”管理体制。

提高开放型经济水平。要把握世界经济发展态势，坚定不移地实施“外向带动”战略。要加大招商引资力度，切实创新招商方式和机制，积极开展产业招商和专业园区招商，着力引进具有较强产业带动力的龙头项目，壮大产业链和产业集群。优化引资和投向结构，把吸收外资与促进产业升级、区域协调发展、国有企业改组改造结合起来。在巩固对港澳台地区招商引资的基础上，进一步开拓欧、美、日等发达国家市场，大力吸引具有行业领先地位的跨国企业集团前来投资。积极推进服务业的对外开放，大力引进生产型现代服务业，鼓励其设立地区总部、研发中心、采购中心，扩大服务出口，发展服务外包。在引进外资的同时，要高度重视引进先进技术、管理经验和高素质人才，切实做好消化吸收和创新提高工作。强化对现有外资企业的服务工作，鼓励外资企业增资扩产、做优做强。充分发挥我市口岸城市和港口资源优势，在保持出口平稳增长的同时，加快转变外贸发展方式，优化出口结构，鼓励自主知识产权和自主品牌的产品出口，提高出口产品质量、档次和附加值。积极扩大进口，重点增加先进技术装备、重要原材料和关键零部件及元器件的进口，不断提高外贸效益和水平。

发展壮大内源型经济。要全面落实促进中小企业发展的相关措施和有关考核办法。拓宽中小企业融资渠道，完善推广“四位一体”融资模式。强化民营中小企业初创代办服务。继续建设面向中小企业的人才培训、市场拓展、信息、技术推广等社会化服务平台和服务体系，大力支持民营经济发展，充分发挥民营经济在促进我市经济发展、社会和谐的生力军作用。

推进大通关建设。要尽快完善口岸发展规划，优化口岸布局。重点推动拱北口岸改扩建工程，加快横琴口岸二期工程客货车通道和“一站式”电子验放系统工程建设，完善珠澳跨境工业区口岸通关功能，推进九洲港口岸改扩建工程建设，加快电子口岸建设试点工作，提高通关效率，共建文明和谐口岸，促进口岸经济发展。

加强珠港澳合作。充分发挥我市毗邻港澳优势，构建珠港澳合作平台，不断拓展合作新领域，提高合作水平，推动珠港澳在基础设施、经贸、旅游、文化教育、环保、民生和应急处理等方面的合作。认真做好横琴岛开发建设控制性详细规划、基础设施建设和项目储备等前期工作，积极争取国家和省的支持，尽快推进横琴岛开发。积极配合做好港珠澳大桥项目规划建设工作，加紧开展大桥对我市经济社会影响和交通对接研究，以更好地发挥大桥对区域经济社会发展的促进作用。年内建成第三条供澳门原水管。加快黄茅岛液化天然气项目建设。促进珠澳跨境工业区产业升级。开展珠澳城市规划、交通设施衔接研究与交流。继续加强珠台经贸技术交流与合作。

（六）着力提高自主创新能力，推进创新型城市建设。

发挥企业自主创新主体作用。要坚定实施“科教兴市”战略，努力营造创新型企业发展壮大、创新型人才施展才华的良好氛围。完善和落实激励自主创新的配套政策，加快建立多元化自主创新投入机制，搭建科技型企业融资平台，引导和鼓励企业加大技术改造和创新力度，加强重点产业、企业、技术中心和工程中心建设，扶持创新型企业尤其是民营科技企业发展，鼓励外贸加工型企业将研发机构迁入珠海，促进自主创新成果产业化发展。

加大创新体系建设力度。加快建立以企业为主体、市场为导向、产业化为目标、高新区和大学园区为依托的科技创新体系。高新区要在提升自主创新能力、加快高新技术产业发展方面充分发挥作用，大力推进自主创新先行示范区建设。引导大学园区高校更好地发挥人才支撑和智力支持作用，支持大学科技园建设，支持建立产学研战略联盟，鼓励、吸引国家重点实验室在珠海设立实验机构，聚集高级人才，围绕我市优势产业开展共性关键技术攻关和技术服务，建立和完善公共技术、公共检测、科技信息和产业集群服务平台，提高我市自主创新能力。

深入实施名牌带动战略。深入推进区域品牌和驰名商标创建工程，鼓励企业创建自有品牌，实施激励自主创新的政府采购政策，奖励荣获国家、省名牌产品和商标企业。加强知识产权保护和专利行政执法，坚决打击盗版行为，支持企业参与国家行业技术标准制定和国际标准化活动，打造更多珠海创造的国家名牌。

大力引进和培养创新型人才。完善人才引进、培养和使用的配套政策，出台人才开发目录，引进和培养一批自主创新的领军人物和中青年高级专家，加快推进以高层次、创新型人才为重点的各类人才队伍建设。加强博士后流动站点建设，鼓励出国留学人员来珠海投资创业。加快人事制度改革，充分激发各类人才的积极性和创造性，营造良好的人才创业环境。

（七）统筹城乡发展，建设社会主义新农村。

推进新农村建设。以城市的理念引导我市西部地区农村发展。坚持以城带乡、以工促农的方针，以发展农村经济、增加农民收入为核心，全面推进新农村建设。要全力推动城市基础设施向农村延伸、城市公共服务向农村拓展、城市社保向农村覆盖、城市文化向农村辐射，形成城乡经济社会发展一体化新格局。要编制新农村建设规划，强化规划在农村建设中的引导作用。继续完善农民特别是被征地农民养老保险制度，扩大农渔业政策性保险试点范围。推进环境优美镇和生态文明村建设。加强农村文化建设，搞好农村和农场就业培训，培育有文化、懂技术、会经营的新型农民。

发展镇村经济。加快中心镇建设，发展各具特色的专业镇。大力推动镇村与园区联动发展，加快推进农村“三化”步伐，促进农村工业向园区集中、农村人口适度向城镇集中、农村土地适度向农业龙头企业集中。大力发展现代农业园区，积极推进台湾农民创业园规划建设，培育和壮大农业龙头企业，鼓励发展农副产品深加工业、生态旅游业和城郊型农业，走特色化、产业化、集约化、市场化发展路子。加强和规范农村股份公司管理。深化农村综合改革，完善责权利相统一的镇街村居管理体制，调动基层的积极性。

改善农村生产生活条件。巩固和完善惠农强农政策，２００８年全市财政一般预算计划安排“三农”投入１０．３亿元，计划从基金预算安排１７．３亿元，共２７．６亿元，同比增长８７％。加大农村道路建设和改造力度，投入８２００万元推进农村路网建设，切实抓好农村危桥、危涵改造，加强低产鱼塘改造和水利防灾减灾工程建设，增强农业生产防灾抗灾能力。建立对水源保护地和农田保护区的生态补偿机制。切实解决农村民生问题，鼓励农民发展农村物业经济或转移就业，增加农民财产性、就业性收入，拓宽农民增收渠道。落实华侨农场改革方案，增强自我发展能力。加大扶贫力度，市安排２０００万元扶持我市年集体纯收入较低的欠发达村和革命老村。完善农民宅基地报建政策，改善村居环境。

（八）继续改善民生，推动和谐珠海建设。

继续加大民生投入。２００８年全市财政一般预算安排教育、医疗卫生、社会保障和就业、文化体育、环境保护、农林水事务等六项支出３５．２亿元，计划从基金预算中安排１７．６亿元，共５２．８亿元，同比增长５５％。新增廉租房建设支出１．４亿元，进一步健全廉租住房制度，加强经济适用住房的建设和管理，构建市民住房保障体系。

推动教育优先发展。全面推进教育现代化建设，促进义务教育均衡发展，加快农村学校规范化建设，做好高中阶段教育发展规划，大力发展职业技术教育。全面实施珠海户籍的中小学生十二年免费教育，新建、扩建一批高中、职业中学，加快城职院一期工程建设。制定促进民办教育和学前教育发展的政策措施，促进民办教育和学前教育发展。全面实施素质教育，加强教师队伍建设。

提高劳动就业和社会保障水平。落实扩面征缴责任制，扩大社会保险覆盖面，不断提高保障能力。深入实施城乡居民基本医疗保险和未成年人医疗保险，建立城乡医疗救助资金。提高低保对象补贴标准，引导低保、残疾等困难群体参加城乡居民基本医疗保险，研究制定低保分类施保机制、自然增长机制和应急补贴机制。健全公共就业服务体系，实施就业培训工程和援助制度。建立帮助零就业家庭等困难群体解决就业的长效机制。坚持实施积极的就业政策，落实以创业带动就业的方针，加强就业和创业培训，鼓励自谋职业和自主创业。今年计划新增就业岗位２６０００个，培训农村青年９０００人，培养高技能人才３６００人。以实施《劳动合同法》和《劳动争议调解仲裁法》为契机，规范和协调劳动关系，依法维护劳动者的合法权益。加强市场调控，稳定市场供应，今年市、区共安排８００万元作为价格调节基金。加大对孤儿、单亲母亲、特困病人、残疾人等社会群体的帮扶力度，发展慈善事业，保障困难群众基本生活。

推进“健康城市”建设。坚持公共医疗卫生等民生事业的公益性质，完善公共卫生服务体系，建立基本医疗卫生制度。扎实开展城区“小病治疗免费”试点。完善免费婚前和孕期医学保健服务制度。办好区级人民医院，建设“卫生强镇”，加快农村“四网合一”的卫生服务中心硬件和软件建设，健全市、区、镇、村四级医疗服务体系。加快建设市疾病预防控制中心，提高重大疾病的防控能力。加强学科人才队伍建设，提高医疗技术和服务水平，为群众提供安全、方便、优质、价廉的医疗卫生服务。以奥运会举办为契机，开展全民健身运动，推进公众健身广场等城乡体育设施建设，办好市运动会，促进体育事业和体育产业发展。

加强社会管理工作。强化“稳定是第一责任”的观念，加强社会治安综合治理，妥善处理历史遗留问题，切实化解社会矛盾纠纷，维护社会稳定。坚持科技强警，加快以社会治安视频监控系统为重点的信息化建设，完善社会治安防控体系，把我市建设成为珠三角社会治安最好的城市之一。构建反走私长效机制，将反走私工作纳入社会治安综合治理考核内容。强化安全生产工作，落实政府安全生产监管责任制和企业安全生产主体责任，开展镇街安全生产执法试点，加大安全隐患排查整治力度，坚决遏制重特大安全事故的发生，确保安全生产形势稳定。完善应急管理体系和工作机制，提高突发公共事件应急处置能力。做好信访、人民调解和法律援助工作，探索建立公益性基层法律服务体系，引导群众合法、合理地表达利益诉求。巩固和扩大产品质量和食品安全专项整治成果，重点清理和整顿“五小”经营单位，推进食品生产加工基地建设，确保食品质量安全。

大力开展文明城市创建活动。积极开展全国文明城市创建工作，大力发展文化事业和文化产业。保障人民基本文化权益，构建文化服务体系，让市民共享文化发展成果。加大镇街、社区和农村文化设施建设的投入，丰富社区、广场和镇村文化生活。支持文艺精品创作，办好珠海首届读书月和金秋艺术节活动。加紧推进市文化馆、数字档案馆等文化基础设施的规划建设。大力发展文化产业，设立文化产业专项扶持资金，加快推动唐家湾历史文化名镇、南方文化产业园和文化创意产业园等项目建设，培育文化产业骨干企业和知名品牌。加大对历史文化名人故居的保护力度，重视保护古村落、古建筑和农村传统特色文化，做好第三次全国文物普查和地情志书编写工作。加强国防动员和双拥共建工作，努力争创广东省“双拥模范城”七连冠。做好第二次全国经济普查工作，充分发挥统计在经济社会发展中的重要作用。继续做好民族宗教、海防、人防、仲裁、气象和港澳流动渔民等工作，支持和保障各人民团体履行职能，充分调动社会各界的积极性，共建和谐社会，共创文明城市。

（九）加快行政管理体制改革，推进服务型政府建设。

积极推进政府管理创新。行政管理体制改革是深化改革的重要内容。要弘扬特区精神，大力推进政府管理创新，着力转变职能、理顺关系、优化结构、提高效能，形成权责一致、分工合理、决策科学、执行畅顺、监督有力的行政管理体制。要深化政府机构改革，促进政府职能转变，在加强和改善经济调节、市场监管的同时，更加注重社会管理和公共服务，努力建设服务型政府。合理配置部门职能，理顺部门职责关系，实行职能有机统一的大部门体制，健全部门间的协调配合机制。完善行政监督制度，实施目标管理、责任白皮书、领导干部任中经济责任审计和行政问责制度，建立符合科学发展观要求的政绩考核办法，形成科学合理，职责、目标、监督、评估、问责一体化的行政责任体系。

加强廉政勤政建设。坚持标本兼治、综合治理、惩防并举、注重预防的方针，扎实推进惩治和预防腐败体系建设。完善政务公开制度，把涉及人民群众切身利益的相关权力运行过程作为政务公开的重要内容。加强制度建设，重点规范财政转移支付、城市规划建设、土地出让开发、政府采购和国有资产转让等公共资源的管理。加强对行政审批权运行的监控，最大限度地减少行政审批的随意性。加大治理商业贿赂工作力度，坚决纠正损害群众利益的不正之风。大力提倡艰苦奋斗，坚决制止奢侈浪费。加强行政监察、政务督查和限时督办，严肃查处有令不行、有禁不止和失职渎职等行为，严肃查处各类违法违纪案件，依法严惩腐败分子，增强政府执行力和公信力。政府公务员特别是领导干部，都要从我做起，从具体事情做起，身体力行，廉洁自律，为全社会做廉洁奉公的表率。

加强民主法制建设。坚持依法行政，推进依法治市，认真落实地方性法规立法计划和“五五”普法规划，加强政府立法，提高立法质量。坚持用制度管权、管事、管人。更加自觉接受人大及其常委会的监督，主动接受人民政协的民主监督，认真听取民主党派、工商联、无党派人士以及各人民团体的意见和建议，虚心接受新闻舆论和社会公众监督。认真执行市人大及其常委会的各项决议，认真研究处理市人大常委会提出的意见，提高人大议案、代表建议和政协提案、建议案的办理质量。完善政府依法、科学、民主决策机制。充分利用市长热线电话和电子邮箱，广泛听取市民各种意见和建议，组织开展为我市科学发展观计献策活动，以开放、包容的态度，畅通倾听民意、集中民智、维护民利的渠道。

建设高素质的公务员队伍。深入开展继续解放思想学习活动，增强公务员推动科学发展的能力和水平。优化选人用人机制，引导公务员争当精通业务的尖兵、干事创业的标兵、推动科学发展的排头兵。加大公务员培训力度，充分发挥行政学院和大学园区高校的作用，加强与外地名校的合作，有针对性地开展专题培训，不断提高公务员的知识水平和行政能力。

各位代表，今年我市工作任务繁重，使命光荣。让我们在省委、省政府和市委的坚强领导下，团结带领全市人民，继续解放思想，坚持改革开放，推动科学发展，为争当实践科学发展观的排头兵而努力奋斗！
